# An epistatic explanation

**DOI:** 10.7554/eLife.21162

**Published:** 2016-09-30

**Authors:** Yoshihiro Komatsu, Yuji Mishina

**Affiliations:** 1 Department of Pediatrics, The University of Texas Medical School at Houston, Houston, United States; 2 Department of Biologic and Materials Sciences, School of Dentistry, University of Michigan, Ann Arbor, United Statesmishina@umich.edu

**Keywords:** craniosynostosis, craniofacial, exome sequencing, human genetics, de novo mutation, incomplete penetrance

## Abstract

Interactions between two gene variants that rarely cause midline craniosynostosis on their own make the development of the disorder a certainty.

**Related research article** Timberlake AT, Choi J, Zaidi S, Lu Q, Nelson-Williams C, Brooks ED, Bilguvar K, Tikhonova I, Mane S, Yang JF, Sawh-Martinez R, Persing S, Zellner EG, Loring E, Chuang C, Galm A, Hashim PW, Steinbacher DM, DiLuna ML, Duncan CC, Pelphrey KA, Zhao H, Persing JA, Lifton RP. 2016. Two locus inheritance of non-syndromic midline craniosynostosis via rare *SMAD6* and common *BMP2* alleles. *eLife* **5**:e20125. doi: 10.7554/eLife.20125

The skull consists of several bony plates that are joined together at cranial sutures, and progenitor cells that reside in these sutures have a crucial role in supporting the rapid growth of new bone to accommodate the growing infant brain. However, in approximately 1 in 2500 babies the cranial sutures fuse together prematurely (either just before birth or shortly afterwards), resulting in a skull deformity known as craniosynostosis (Wilkie et al., 2010). In this condition, the bones of the skull cannot grow quickly enough to keep up with the growing brain, resulting in increased pressure inside the skull. If craniosynostosis is left untreated, it can result in a loss of vision, hearing problems and chronic headaches.

Although some cases of craniosynostosis have been linked to a number of genetic mutations, 70% of observed cases still have no known genetic cause (Twigg and Wilkie, 2015). A form of craniosynostosis called non-syndromic midline (metopic and sagittal) craniosynostosis accounts for half of all reported cases (Greenwood et al., 2014; Figure 1), but very few of these cases have been associated with genetic mutations. Now, in eLife, Richard Lifton and colleagues at Yale University School of Medicine and Craniosynostosis and Positional Plagiocephaly Support, Inc. – including Andrew Timberlake as first author – report that two genetic mutations, each of which has little impact on its own, can result in devastating outcomes when they are both present (Timberlake et al., 2016).

**Figure 1.**
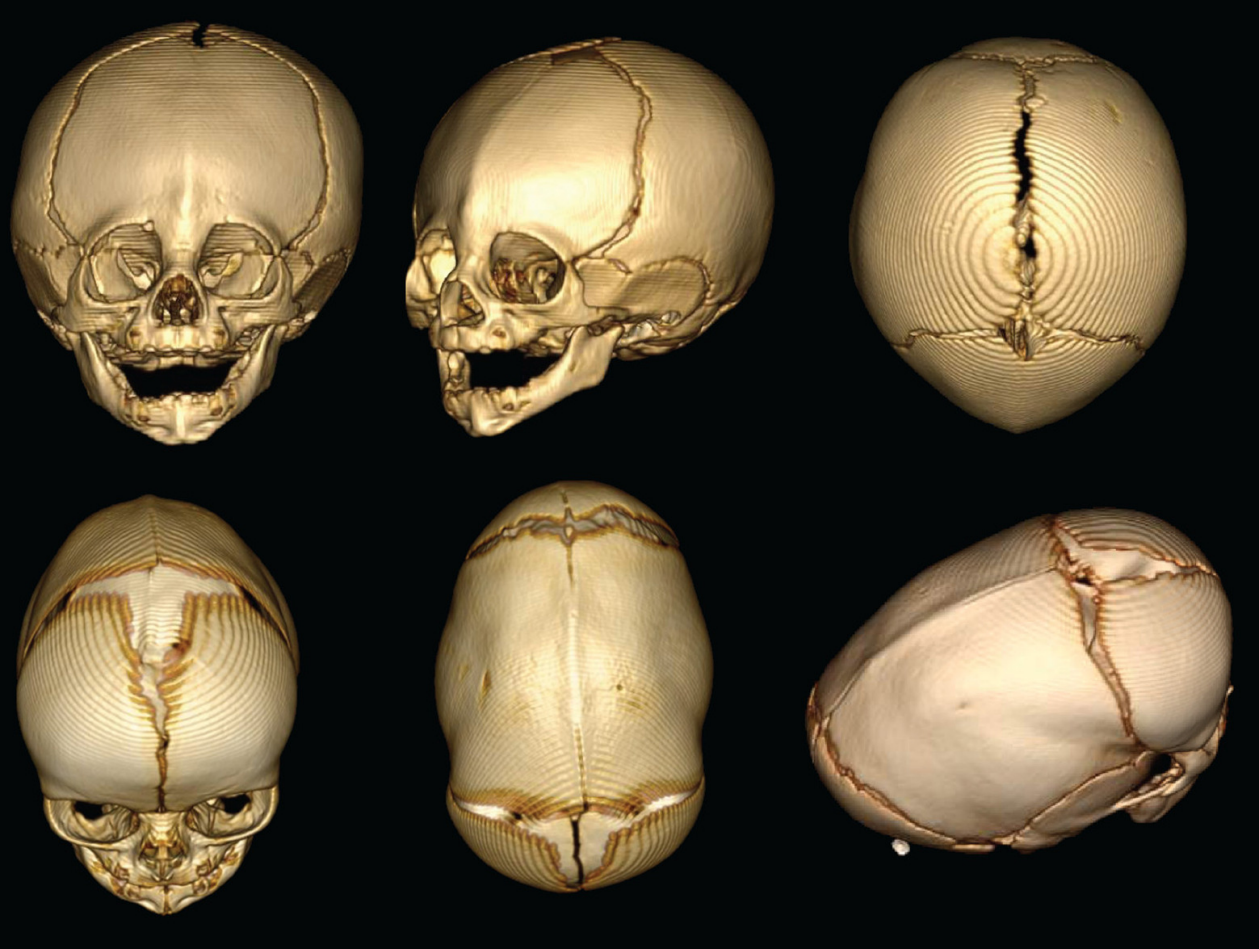
3D reconstructions of the skull shapes that result from two types of non-syndromic craniosynostosis. Top row: In metopic craniosynostosis, the metopic suture that passes down the middle of the forehead fuses prematurely. This results in a narrow forehead that forms with a ridge down the suture. In an attempt to compensate, the skull grows in a way that pushes the forehead forward. Bottom row: sagittal craniosynostosis results from the premature fusion of the sagittal suture along the top of the skull. As a result, the skull stays narrow and extends toward the front and back to compensate.

Genome-wide association studies have identified several single nucleotide polymorphisms that are associated with non-syndromic midline craniosynostosis (Justice et al., 2012). The most significant of these mutations is located close to the gene for a bone morphogenetic protein (BMP) called BMP2, so this site might act as an enhancer to increase the production of BMP2 (Justice et al., 2012). BMP is known to be important in bone development (Urist, 1965), and previous studies have suggested that altered BMP signaling leads to craniosynostosis (Jabs et al., 1993).

It has been suggested that the sporadic occurrence of non-syndromic craniosynostosis is due to de novo (non-inherited) genetic mutations and/or because these mutations do not consistently produce the same symptoms. To identify genetic mutations associated with midline craniosynostosis, Timberlake et al. sequenced the whole exome (that is, the part of the genome formed by exons) of 191 people who were the first in their family to display the symptoms of non-syndromic midline craniosynostosis; they also sequenced the exomes of both parents of 132 of these volunteers. The sequences revealed a number of different de novo mutations in the gene that encodes SMAD6, a molecule that is known to inhibit BMP signaling. Most of the mutations prevent the SMAD6 protein from working.

Timberlake et al. found that *SMAD6* mutations produce craniosynostosis in only 9% of cases. However, when an individual has both the *SMAD6* mutation and the *BMP2* risk allele (which on its own leads to the disorder in just 0.08% of cases), craniosynostosis occurs 100% of the time. Thus, these findings clearly indicate that epistatic interactions – interactions where the effect produced by a given gene depends on the presence of other genes – of rare (*SMAD6*) and common (*BMP2*) gene variants are a defining feature of the cause of midline craniosynostosis. As a result, these genetic mutations are likely to account for as many cases of craniosynostosis as the single mutation in a gene called *FGFR2* that is currently known as the most frequent cause of craniosynostosis.

Timberlake et al. also show that there is no epistatic interaction between the mutant *SMAD6* variant and the other mutation that is known to be associated with midline craniosynostosis (a single nucleotide polymorphism in *BBS9*; Justice et al., 2012). This finding further supports the hypothesis that an increase in BMP signaling is likely to underlie the symptoms of craniosynostosis.

BMPs can cause bone to form in abnormal places (Urist, 1965), so it is possible that the symptoms of craniosynostosis are caused by increased bone formation "filling in" the cranial suture and depleting the numbers of progenitor cells found there. Bone forms as a result of these osteoprogenitor cells developing into cells called osteoblasts, which secrete the materials that form bone. However, the story may not be so simple, because if increased BMP signaling occurs only in osteoblasts, mice do not develop the symptoms of craniosynostosis (Komatsu et al., 2013). Instead, an increased rate of cell death in the osteoprogenitor cell population seems to be the primary cause of craniosynostosis in mutant mice that exhibit excessive BMP signaling. To support this notion, mutant mice treated in the womb with drugs that prevent cell death do not develop skull deformities (Hayano et al., 2015). The obvious next steps are to understand the cellular mechanisms that cause cranial sutures to fuse prematurely, and to confirm whether human patients with mutations in the *Smad6* and *BMP2* genes experience increased cell death in the sutures.

At present the only option for treating craniosynostosis is repeated reconstructive surgery: however, the work of Timberlake et al. and other groups suggest that an alternative therapeutic approach could be to prevent cell death in sutures. Developing such an approach will require researchers to pool together all the knowledge that has been gained from a wide range of genetics studies in both mice and humans. This is therefore a crucial moment in the search for the best treatment strategy for a large proportion of people with non-syndromic craniosynostosis.
